# Comparison of mechanisms of reproductive interference in *Taraxacum*

**DOI:** 10.1093/aob/mcz007

**Published:** 2019-02-02

**Authors:** Akane Takemori, Akiyo Naiki, Ko-Ichi Takakura, Masahiro M Kanaoka, Sachiko Nishida

**Affiliations:** 1 Graduate School of Education, Okayama University, Kita-ku, Okayama City, Japan; 2 Iriomote Station, Tropical Biosphere Research Center, University of the Ryukyus, Yaeyama-gun, Japan; 3 School of Environmental Science, The University of Shiga Prefecture, Hikone, Japan; 4 Graduate School of Science, Nagoya University, Chikusa-ku, Nagoya, Japan; 5 Nagoya University Museum, Chikusa-ku, Nagoya, Japan

**Keywords:** Competition for pollination, heterospecific pollen deposition, hybridization, pollinator preference, reproductive interference, *Taraxacum officinale*, *Taraxacum japonicum*

## Abstract

**Background and Aims:**

Reproductive interference may reduce fitness of either of the involved species, with potentially important ecological and evolutionary consequences. Except for the effect of shared pollinators on reproductive success, however, mechanisms underlying reproductive interference have been little studied, even though the severity of its impact may depend on the specific mechanism. The aim of this study was therefore to explore the mechanisms of reproductive interference between *Taraxacum japonicum* (native to Japan) and *Taraxacum officinale* (alien).

**Methods:**

In a field survey, the association between alien species density and seed set in *T. japonicum*, and whether pollinator behaviour indicated a preference for the alien, were examined. Effects of heterospecific pollen deposition were measured in a series of hand pollination experiments, including mixed pollination experiments in which the order of application of conspecific and heterospecific pollen was varied. Finally, to investigate hybridization frequency, the parentage of seedlings produced following natural, mixed or heterospecific pollination was compared.

**Key Results:**

Alien species density did not negatively affect native seed set, nor did pollinators appear to have a preference for alien flowers. The hand pollination experiments showed that heterospecific pollen deposition adversely affected native seed set, especially when alien pollen was applied before conspecific pollen. No viable hybrids were found following natural pollination, which suggests that hybridization might be a rare event.

**Conclusion:**

Among the examined mechanisms, heterospecific pollen deposition might have the largest deleterious effect on the native species. This effect is frequency dependent; thus, a positive feedback loop may cause the effect on the population dynamics to increase over time, with the result that the alien might eventually displace the native in a population. Effects of the examined mechanisms on population dynamics should be investigated further to improve understanding of the impact of reproductive interference on the structure of plant communities.

## INTRODUCTION

Reproductive interference, defined as a negative effect of interspecific sexual interaction on the fitness of either species, is an important mechanism that can explain patterns of exclusion among closely related species (Hochkirch *et al.*, 2007; Gröning and Hochkirch, 2008). In plants, reproductive interference is often synonymous with competition for pollination, especially for pollination by animals. Competition for pollination is defined by Waser (1983) as any interaction in which co-occurring plant species (or phenotypes) suffer reduced reproductive success because they share pollinators. Temporal, spatial and morphological impacts of competition for pollination on the structure of plant communities have received much attention since their importance was first recognized by Robertson (1895) (Mitchell *et al.*, 2009).

With the exception of the effect of shared pollinators on reproductive success of the plant species involved, however, the mechanisms underlying reproductive interference have been less studied, even though Arceo-Gómez and Ashman (2011) have reported that the severity of its impact, as well as its ecological or evolutionary consequences, depends on the specific mechanism involved. Pollinator sharing can cause a reduction of seed set in the focal species by two processes: by reducing conspecific pollen transfer, which may cause a pollen limitation (conspecific pollen loss), or by increasing heterospecific pollen deposition, which may interfere with reproduction of the focal species (e.g. Waser, 1978; Caruso and Alfaro, 2000; Bjerknes *et al.*, 2007; for reviews, see Morales and Traveset, 2008; Mitchell *et al.*, 2009). The decrease in conspecific pollen transfer is expected to become more severe as the density of the counterpart species increases, whereas an increase in the density of the counterpart species should not affect heterospecific pollen deposition unless its frequency in the total population also increases (Takakura *et al.*, 2009; Nishida *et al.*, 2017). In addition, conspecific pollen loss and heterospecific pollen deposition would have opposite results, depending on the abundance of pollinators. A model by Campbell (1986) predicts that the relative intensity of competition will diminish as the pollinator visit rate increases if the competition occurs by conspecific pollen loss due to interspecific pollen movement, whereas fruit set may drop even when pollinators are very abundant if the stigma suffers from foreign pollen interference. Because of these differences, conspecific pollen loss and heterospecific pollen transfer might have different consequences for the population dynamics, community assemblage and evolution of the reproductive strategy (e.g. Harder and Aizen, 2010; Eaton *et al.*, 2012). Studies that have examined these mechanisms (e.g. Thomson *et al.*, 1981; Waser and Fugate, 1986; Harder *et al.*, 1992; for a review, see Morales and Traveset, 2008) have contributed to a constructive discussion of the generality and uniqueness of each mechanism with respect to their ecological and evolutionary significance.

In this study, we explored in detail the mechanism of reproductive interference between an alien and a native species of *Taraxacum*. The alien species, *T. officinale*, has been reported to sometimes displace its native congener, *T. japonicum*, and reproductive interference from the alien to the native species may be responsible for this displacement (Takakura *et al.*, 2009; Nishida *et al.*, 2017). Both field surveys and hand pollination experiments have shown that the strength of reproductive interference from the alien to the native *Taraxacum* species in Japan is related to observed displacement trends: severe reproductive interference from the alien to the native species is found where the native species has been largely displaced by the alien species, whereas where the natives have low sensitivity to reproductive interference the aliens and natives coexist (Takakura *et al.*, 2009; Matsumoto *et al.*, 2010; Nishida *et al*., 2012, 2017). Nishida *et al.* (2014) reported that ovule usurpation following heterospecific pollen deposition is the primary mechanism of reproductive interference in *Taraxacum*, although other mechanisms, including pollinator preference (Kandori *et al.*, 2009) and hybridization (Mitsuyuki *et al.*, 2014), have also been reported to negatively affect the native population dynamics.

The purpose of this study was to evaluate these previous findings and clarify the actual mechanisms of reproductive interference from an alien to a native species of *Taraxacum* by conducting field surveys, hand pollination experiments and genetic analyses of seedlings. We first examined the relationship between alien species density and seed set of the native species in light of a prediction by Feinsinger *et al.* (1991) that they would be negatively correlated if competition for pollinators or conspecific pollen loss by the shared pollinators severely affected the native’s reproductive success. We also observed pollinator behaviour to determine whether a pollinator preference for the alien flowers was present. In a series of hand pollination experiments of native flowers, including staggered mixed pollination experiments in which the order of application of heterospecific and conspecific pollen was varied, we examined the effects of heterospecific pollen deposition on seed set. Finally, we compared the parentage in viable seedlings produced following natural pollination and our hand pollination experiments to investigate whether the threat to native populations of hybridization was similar to that of other mechanisms. From the results of these examinations and evaluations, we inferred the mechanisms that critically affect the reproductive success of the native species in the studied populations.

## MATERIALS AND METHODS

### Study species

*Taraxacum japonicum* is a diploid dandelion species (2*n* = 16) that is native to the lowlands of western Japan. Diploid dandelions reproduce sexually and are self-incompatible (Richard, 1970). The alien congener, *T*. *officinale*, is native to Europe and is now distributed throughout Japan (Ogawa and Mototani, 1985; Hoya, 2010). In Japan, *T. officinale* is polyploid and agamospermous (Morita, 1980). The native diploid species and pure *T. officinale* are easily differentiated in Japan by the morphology of the involucre (bracts holding a capitate inflorescence): in *T. japonicum*, the outer involucre bracts are appressed, whereas in *T. officinale*, they are strongly reflexed. Although it is mainly agamospermous in Japan, triploid *T. officinale* produces pollen and hybridizes with native dandelions (Shibaike *et al.*, 2002). In this study, we counted hybrids with the alien species, both because many putative alien dandelions in western Japan are actually hybrids (Shibaike *et al.*, 2002) and because all previous studies on reproductive interference in *Taraxacum* have included the hybrids with the alien species (e.g. Takakura *et al.*, 2009; Matsumoto *et al.*, 2010; Nishida *et al*., 2012, 2017). Like *T. officinale*, hybrids between *T. officinale* and *T. japonicum* are agamospermous (Hoya, 2010). Some hybrids in Japan are tetraploid and lack pollen grains (Hoya, 2010), but the tetraploid hybrids tend to bloom later than other *Taraxacum* species in Japan (K.I. Takakura and S. Nishida, pers. obs.). All of the plants identified as hybrids in this study had pollen grains and were assumed to be triploid.

### Study sites

We conducted this study at two sites, HB (Handa City Botanical Garden; 34°53′34″N, 136°56’17’’E) and OU (Okayama University; 34°41′18″N, 133°55′20″E), in Okayama, Japan, in the central part of the distribution range of *T. japonicum*. The two sites are about 1.5 km apart. HB is along roads in a botanical garden that is surrounded by secondary forests composed mainly of *Quercus acutissima*, *Cerasus jamasakura* and *Acer palmatum*, and pollinators are presumably abundant. OU is a mostly open grassland site on a university campus that is surrounded by cultivated trees and shrubs, and pollinators are expected to be less abundant.

### Field survey of the association between alien density and native reproductive success

A field survey was carried out at our two study sites on 1 May 2012 and 16 April 2014. We arbitrarily selected 10–24 native individuals as sample plants, and counted the number of native and alien inflorescences (flower heads) within a 2 m radius of each sample plant. The inflorescence has been found to be the most reasonable counting unit in surveys of density-dependent effects (e.g. Kandori *et al.*, 2009; Takakura *et al.*, 2009) because pollinators usually move from inflorescence to inflorescence and pollinate most of the flowers in each *Taraxacum* inflorescence during a single visit. The radius of 2 m was adopted because Takakura *et al.* (2011) previously showed that to be the effective distance for pollen transfer from the alien to the native species. About 2 weeks after each survey, once the flowers of the sample plants were observed to have developed fruit, we collected one infructescence from each sample plant and counted the number of developed and undeveloped ovules in each infructescence. We defined seed set as the ratio of normally developed ovules to the total number of achenes in each infructescence. A single *Taraxacum* inflorescence comprises a variable number of flowers, from about 30 to 90, and each flower contains only one ovule, which becomes an achene. Since achenes remain on the infructescence until dispersal, whether the ovules develop successfully or not, we could determine seed set by counting the developed and undeveloped ovules in each infructescence. Following Nishida *et al.* (2012), we considered achenes with a hard, brown pericarp to be successfully developed and those with a soft, white pericarp to be undeveloped. We used a generalized linear mixed model (GLMM; Wolfinger and O’Connell, 1993) with a binomial error structure and a logit link function to analyse the association between alien density and native seed set. The response variable was the development of the sampled ovules (developed or undeveloped), and the explanatory variables were the numbers of alien and native inflorescences within a 2 m radius of the sample plant. We considered the number of alien inflorescences to reflect the density-dependent aspect of the pollinator preference for aliens, and the number of native inflorescences to reflect the intraspecific facilitation of pollination as well as competition for pollinators among the natives. We did not use the relative abundance of the alien inflorescences (ratio of the alien inflorescences to total inflorescences), because that ratio reflects frequency-dependent effects rather than density-dependent effects. Both the frequency and the density of the alien inflorescences influence pollinator behaviour, but use of the alien inflorescence frequency in the analysis would mean that the effects of alien abundance would not be evaluated. The individual sample plants were treated as a random effect. R version 3.0.3 (R Core Team, 2014) was used for the GLMM analyses. We considered the effects of alien density and native density on native seed set to be significant if the *P*-value was <0.05.

### Observation of pollinator visitation

We examined whether there was a pollinator preference for alien inflorescences by observing pollinator visitations at HB and OU on 12 and 13 April 2018, when both *Taraxacum* species were in full bloom. We arbitrarily selected 18 spots at HB and 12 spots at OU, each with a radius of 2 m, and counted the number of native and alien inflorescences in each spot. We then observed pollinator visitations for 30 min at each spot and recorded the taxa of the visitors, the *Taraxacum* species that they visited and the order of their visitations (from native to native, from native to alien, from alien to alien or from alien to native inflorescence) while at the spot. To prevent time of day from being a factor in our analysis, we rotated the order of observation at the two study sites; we observed pollination at OU in the morning of 12 April and in the afternoon of 13 April, whereas at HB we observed pollination in the afternoon of 12 April and in the morning of 13 April (both days were similarly sunny). To prevent the observer from being a factor, two people carried out observations at different spots during each observation period: one person observed a spot with more native species and the other person observed a spot with more alien species; then, during the next observation period, the roles of the two people were exchanged. To prevent the same pollinator individuals from being counted by both observers, the spots being observed during the same period were usually at least 20 m apart or separated by some barrier. To assess pollinator preference, we used a model basically similar to that developed by Manly *et al.* (1972) for assessment of predator preference, which is suitable to use when exploitation is non-negligible (Cock, 1978). A brief explanation of our model follows.

If an alien population A and a native population B in a study plot have *a* and *b* inflorescences, respectively, then the logit of *p*, the proportion of inflorescences of population A relative to the total number of inflorescences, is calculated as follows:

$$\begin{array}{l} logit\left(\;p\right)=\log\frac{p}{1-p}=\log\frac{\frac{a}{a+b}}{1-\frac{a}{a+b}}=\log\frac{a}{b} \end{array}$$

**Fig 1.**
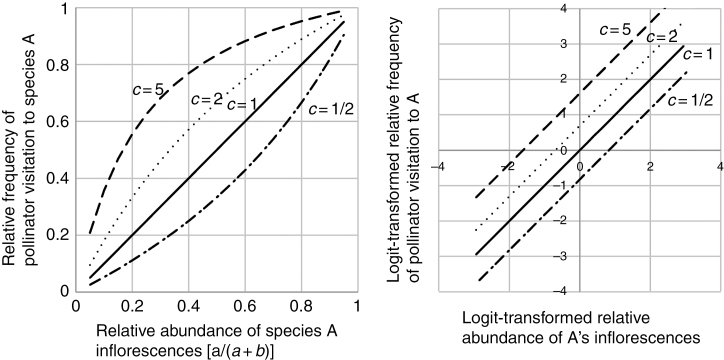
Examples of regression curves (left graph) and logistic regression lines (right graph) calculated by using different values of pollinator preference *c*. When *c* = 1/2 (dash-dot lines), pollinators visited inflorescences of population B twice as many times as those of A, and when *c* = 1 (solid lines), pollinators showed no preference between inflorescence of populations A and B. When *c* = 2 (dotted lines), pollinators visited inflorescences of A twice as many times as those of B, and when *c* = 5 (dashed lines), they visited inflorescences of A five times more often than they visited those of B. The intercept of the logistic regression line is equal to the logarithm of *c*: log(1/2) = –0.693; log(1) = 0; log(2) = 0.693; and log(5) = 1.61.

If a pollinator visits inflorescences of population A *c* times more often than it visits those of population B (see left graph of Fig. 1 for examples), then the logit of *q*, the proportion of visitations to A among total visits, is calculated as follows:

$$\begin{array}{ll} logit\left(q\right) & =\log\frac{q}{1-q}=\log\frac{\frac{ca}{ca+b}}{1-\frac{ca}{ca+b}}\\ & =\log\frac{a}{b}+\log c=logit\left(\;p\right)+\log c \end{array}$$

Therefore, in a logistic regression analysis in which the number of visitations to inflorescences of population A is the response variable, the logit-transformed proportion of inflorescences of A in the total is the explanatory variable and the coefficient of the explanatory variable is set to 1, the intercept should be the logarithm of the preference *c* (log *c*) (see right graph of Fig. 1 for examples). This framework can also be applied to more complex model structures, such as that of the GLMM used in this study.

Using this model, we can estimate that if the intercept is >0, then the pollinators preferred population A (alien) inflorescences to those of B (native); if the intercept is 0, the pollinators did not have any preference; and if the intercept is <0, then the pollinators preferred inflorescences of B to A. We used R version 3.0.3 (R Core Team, 2014) for the analyses and considered the pollinator’s preference to be significant if the *P-*value was <0.05.

### Examination of heterospecific pollen deposition

To evaluate the effects of heterospecific pollen deposition on native seed set, we conducted a series of hand pollination experiments in the population at OU in 2012, 2013 and 2014, and in the population at HB in 2013. We basically followed the methodology used by Matsumoto *et al.* (2010) and Nishida *et al.* (2012). In this series of experiments, we arbitrarily selected 20–38 individuals of the native species at each study site as recipients and assigned the inflorescences of each individual to one of the following two treatments: (1) conspecific pollination with pollen of the native species; and (2) pollination with a mixture of native and alien pollen (mixed pollination) deposited simultaneously. In 2015, we performed a second series of experiments at OU. In these experiments, we followed the same procedures as in the other years and added two more treatments: (3) mixed pollination with the native pollen deposited before the alien pollen; and (4) mixed pollination with the alien pollen deposited before the native pollen. For the experiment in 2015, we used a total of 20 native individuals, but some of them lacked sufficient inflorescences for all of the treatments, so 10–15 individuals were used for each treatment. For all hand pollinations conducted from 2012 to 2015, we used inflorescences that had opened in the morning and we began to hand-pollinate them before any pollinators had visited. As pollen donors, we collected native and alien *Taraxacum* inflorescences from at least 100 m away from the study sites; we applied the donor pollen to the recipient inflorescences directly, by gently touching the recipient stigmas with the pollen grains that were accumulated at the top of the donor flowers. We did not count the number of pollen grains applied, but we used one donor inflorescence to hand-pollinate one recipient inflorescence. The amount of pollen thus applied might be more than sufficient compared with natural pollination, but we chose this amount to avoid the risk that a conspecific pollen limitation might skew our results, and to standardize the amount of pollen from each donor among the treatments. We used each donor inflorescence only once; for the next recipient, we used a different inflorescence to avoid contamination. In the simultaneous mixed pollination experiment (treatment 2), we applied the native pollen first, immediately followed by the alien pollen, as described by Matsumoto *et al.* (2010). In the staggered mixed pollination treatments 3 and 4, we first applied conspecific (experiment 3) or alien (4) pollen, and then about 4 h later we applied alien (3) or native (4) pollen to each recipient. We set the interval between pollinations to 4 h because *Taraxacum* inflorescences at our study sites usually bloomed from around 09.00 to 14.00 h local time; therefore, we assumed that the stigmas might accept pollen over a period of around 5 h. Because florets of *Taraxacum* in our study sites opened sequentially over 2 d (florets of outer whorls opened in the first morning and those of inner whorls opened in the second morning), we repeated the same procedure on 2 d and completed the pollinations to all the florets in each recipient inflorescence. Before and after each hand pollination, we covered the recipient inflorescence with a polyester net to prevent unintended pollination by insects. After the pollinated recipient flowers developed fruits, we collected the infructescences and counted the number of developed and undeveloped ovules in each infructescence to determine the seed set, using the same definition of seed set and distinguishing between developed and undeveloped ovules in the same way as in the field survey. We analysed the association between each treatment and native seed set by using a GLMM with a binomial error structure and a logit link function. The response variable was the development of the sampled ovules (developed or undeveloped), and the explanatory variable was the treatment: conspecific pollen only (treatment 1) or one of the mixed pollination treatments (treatment 2, 3 or 4). The individual was treated as a random effect. We used R version 3.0.3 (R Core Team, 2014) for the analysis. We considered the effect of mixed pollination on seed set to be significant if the *P*-value was <0.05.

### Genotyping by allozyme variation

Pure native *T. japonicum* and hybrids between the native species and alien *T. officinale* can be distinguished by examining the banding pattern of the aspartate aminotransferase enzyme (AAT; EC 2.6.1.1) (Morita, 1997). Therefore, to estimate the frequency of hybridization between the native and alien species producing viable seeds, we determined the AAT genotype of each seedling produced by natural, mixed or heterospecific pollination of *T. japonicum*. To obtain seedlings produced by the natural pollination, we arbitrarily selected 16 *T. japonicum* individuals growing around OU in April 2014, collected one infructescence from each individual and extracted its seeds. In the field where we collected the infructescences, about 35 % of *Taraxacum* belonged to the alien species. We also collected seeds from the mixed pollination experiment conducted at OU in 2014 (see ‘Examination of heterospecific pollen deposition’ for details), and we conducted a heterospecific pollination experiment (hand pollination with alien pollen only) of *T. japonicum* and collected the resulting seeds (seed set was 13.8 %). We sowed 621, 740 and 190 seeds from the natural, mixed and heterospecific pollinations, respectively, on sterilized expanded vermiculite maintained at a temperature of about 20 °C, and 264, 304 and 96, respectively, of the seeds germinated (average germination rates 47.3, 49.4 and 57.6 %, respectively). About 3 weeks later, we collected a piece of fresh leaf (about 100 mg) from each surviving seedling. We eventually obtained 255 leaf samples (from 16 mothers) from the natural pollination, 224 samples (from 16 mothers) from the mixed pollination and 96 samples (from 15 mothers) from the heterospecific pollination. We conducted electrophoresis experiments, basically following the methodology of Shiraishi (1988), using vertical discontinuous polyacrylamide slab gels. We crushed each sample in 1 mL of extraction liquid, transferred it into a 1.5 mL tube and centrifuged it at 17 335 *g* for 60 min. The supernatant liquid was then transferred into a new 1.5 mL tube and stored at –80 °C until analysis. Electrophoresis was conducted with a constant current of 15 mA cm^–2^ for about 150 min. Staining procedures followed those used by Richardson *et al.* (1986), Shiraishi (1988) and Tsumura *et al.* (1990). Besides the seedlings, we checked AAT genotypes of 30 alien individuals arbitrarily collected at OU, and confirmed that none of the examined aliens had a similar banding pattern to that of the surrounding *T. japonicum*. We also determined the banding patterns of all pollen donors used for either conspecific or heterospecific pollination in the hand pollinations, and used only donors with a different banding pattern from that of the recipient for each treatment. For the seedlings produced by the natural pollination, we observed their banding patterns and identified a sample as from a pure native species if it had only the pattern of the surrounding native species, or as a hybrid if it had patterns of both the native and alien species. In the same way as for the seedlings produced by the hand pollinations, we identified a sample as from a pure native species if it had the combined banding patterns of the native mother and the native pollen donor, or as from a hybrid if it had the combined banding patterns of the native mother and the alien pollen donor.

## RESULTS

### Field survey of the association between alien density and native reproductive success

**Fig 2.**
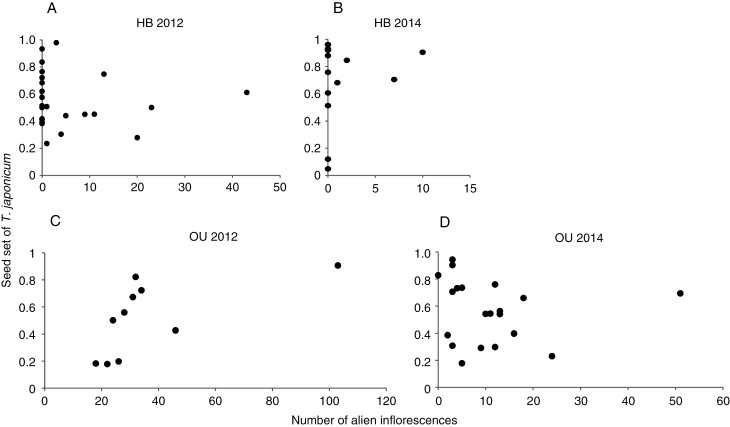
Relationships between the density of alien *Taraxacum* (number of alien inﬂorescences within a 2 m radius of each *T. japonicum* sample plant) and seed set (ratio of the number of normally developed seeds to the total number of achenes) of *T. japonicum* at HB (A, B) and OU (C, D) in 2012 (A, C) and 2014 (B, D).

Seed set of the native species did not decrease significantly as the number of surrounding alien inflorescences increased, which suggests that alien density did not negatively affect the reproductive performance of the native species (Table 1; Fig. 2). In fact, native seed set increased significantly when the natives were surrounded by more alien inflorescences at OU in 2012, although this association was not strong (coefficient ± s.e. = 0.040 ± 0.012). As for the number of native inflorescences, its effect on native seed set was not significantly negative but was once weakly positive at OU in 2014. This result implies that the reproductive performance of the native species was usually independent from or weakly facilitated by abundant conspecific flowers.

**Table 1.** GLMM analysis results for the effect of alien and native density (number of alien and native inflorescences within a 2 m radius) on seed set in native *Taraxacum japonicum*

Locations	Year	*n*	Effect of alien inflorescence density			Effect of native inflorescence density		
			Coefficient ± s.e.	*Z*	*P*	Coefficient ± s.e.	*Z*	*P*
HB	2012	24	0.014 ± 0.024	0.578	0.563	0.017 ± 0.009	1.847	0.065
	2014	13	0.079 ± 0.151	0.525	0.599	–0.011 ± 0.014	–0.807	0.42
OU	2012	10	0.040 ± 0.012	3.299	<0.001	–0.025 ± 0.014	–1.719	0.086
	2014	20	–0.004 ± 0.021	–0.175	0.861	0.063 ± 0.031	2.02	0.043

### Observation of pollinator visitations

Both the major pollinator taxa and the *Taraxacum* species that the pollinators tended to visit were similar at the two locations (Table 2). *Andrena knuthii* (Hymenoptera) was the visitor most frequently observed at both locations (52.7 and 49.1 % of all visitors at HB and OU, respectively), followed at HB by *Andrena* sp. (11.5 %), *Tetralonia nipponensis* (Hymenoptera, 10.8 %), a Diptera species (7.9 %) and a Syphoidae species (Diptera, 6.1 %), and at OU by a Syphoidae species (18.1 %), *T. nipponensis* (6.9 %) and *Nomada okubira* (Diptera, 6.9 %).

**Table 2.** Pollinator visits to native (*Taraxacum japonicum*) and alien (*T. officinale*) inflorescences

Location	*Taraxacum* species	No. of visits by insect order			Total
		Hymenoptera	Diptera	Others	
HB (18)*	Native	169	32	2	203
	Alien	55	16	5	76
OU (12)*	Native	55	22	6	83
	Alien	19	10	4	33
Total		298	80	17	395

*Total number of spots observed.

Many of the pollinators visited only native inflorescences, and rather lower percentages of the visitations were made to aliens or to both natives and aliens (Table 3). When the coefficient was fixed at 1, the intercept of the logistic regression (log *c*), which indicates the pollinator preference for alien inflorescences (see the Materials and Methods for details) was less than zero (–8.80 and –8.94 at HB and OU, respectively; Table 3). This result suggests that the pollinators actually preferred the native inflorescences over the alien inflorescences.

**Table 3.** Pollinator visits to native or alien species and pollinator movements between native (*Taraxacum japonicum*) and alien (*T. officinale*) species, and logarithm of the pollinator preference *c* for the alien species

Location	Percentage of total visitations			Percentage of total movements*					Preference for aliens	
	*n*	Native (%)	Alien (%)	*n*	Native to native (%)	Alien to alien (%)	Native to alien (%)	Alien to native (%)	Log *c*	*P*
HB	279	72.8	27.2	98	66.3	9.2	15.3	9.2	–8.800	<0.001
OU	116	71.6	28.4	42	76.2	4.8	9.5	9.5	–8.940	<0.001

See the text for the model.

*Visitations to only one inflorescence in a spot were excluded from these calculations.

### Examination of the effect of heterospecific pollen deposition

**Fig 3.**
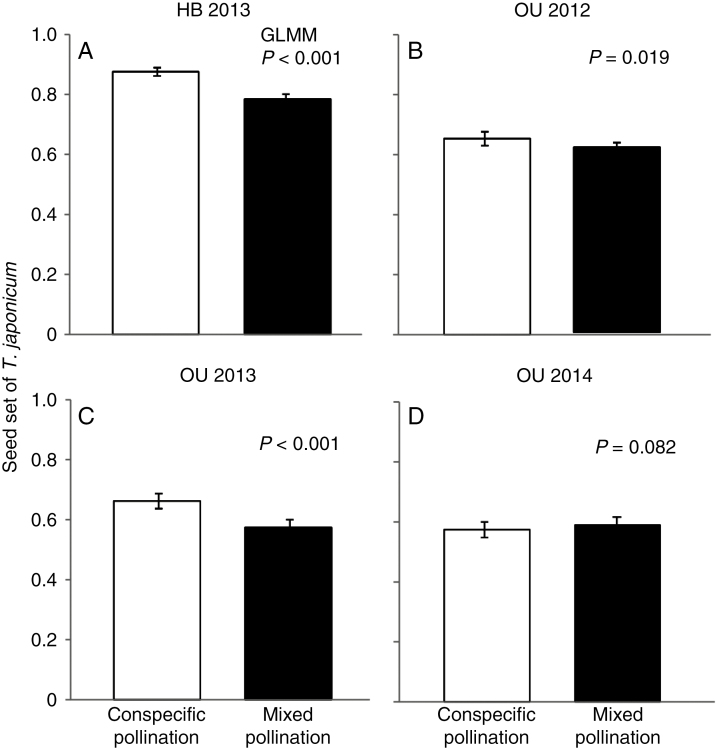
Comparison of seed set (ratio of normally developed seeds to the total number of achenes) in native *Taraxacum japonicum* between conspecific pollination (white bars) and mixed pollination (pollination by both conspecific and alien pollen grains, black bars) at (A) HB in 2013 and (B–D) OU in 2012–2014, respectively. Error bars show 95 % confidence intervals.

**Fig. 4.**
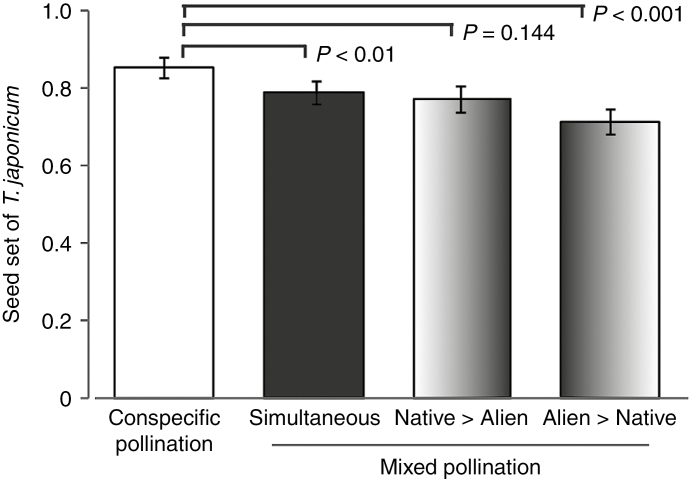
Comparison of seed set in native *Taraxacum japonicum* between conspecific pollination and mixed pollination when the order of application was varied: simultaneous pollination, application of native followed by alien pollen, and application of alien followed by native pollen. Error bars show 95 % confidence intervals.

In three of the four hand pollination experiments conducted from 2012 to 2014, seed set of the native species was significantly lower following mixed pollination than following conspecific pollination (Fig. 3; Table 4). These results indicate that the addition of alien pollen often had a negative effect on the reproductive performance of the native plants. In the mixed pollination experiments in which the order of pollen application was varied, native seed set was significantly lower when the alien pollen was applied simultaneously with or earlier than the native pollen, whereas when the conspecific pollen was applied earlier than the alien pollen, seed set did not differ significantly from that of conspecific pollination only (Fig. 4; Table 5). Seed set was lowest when alien pollen was applied earlier than conspecific pollen. Thus, heterospecific pollen deposition adversely affected native seed set, especially when the alien pollen was deposited before conspecific pollen.

**Table 4.** GLMM analysis results comparing the native seed set of *Taraxacum japonicum* after mixed pollination with that after conspecific pollination

Location	Year	*n*	Average percentage of developed seeds in all ovules		Coefficient ± s.e.	*Z*	*P*
			Conspecific pollination (%)	Mixed pollination (%)			
HB	2013	38	87.6	78.4	–0.729 ± 0.085	–8.61	<0.001
OU	2012	25	65.3	61.7	–0.184 ± 0.078	–2.347	0.019
OU	2013	22	66.2	57.4	–0.428 ± 0.087	–4.927	<0.001
OU	2014	20	57.4	56.6	0.162 ± 0.093	1.74	0.082

**Table 5.** Results of GLMM analysis results comparing native (*Taraxacum japonicum*) seed set after mixed pollination in a different order with that after conspecific pollination at OU in 2015

Pollination	Order	*n*	Average percentage of developed seeds in all ovules (%)	Coefficient ± s.e.	*Z*	*P*
Conspecific	–	12	83.4	–		–
Mixed	Simultaneous	15	75.3	–0.528 ± 0.162	–3.248	0.001
Conspecific first	10	76.0	0.325 ± 0.222	1.463	0.144
	Heterospecific first	13	69.8	–0.594 ± 0.161	–3.688	<0.001

### Genotyping by allozyme variation

Our examination of AAT allozyme variations did not identify even one hybrid seedling produced by natural pollination (Table 6). Of the seedling produced by (simultaneous) mixed pollination and heterospecific pollination, 7.1 and 10.4 %, respectively, were hybrids (Table 6). By multiplying these results by the average seed sets (56.6 and 13.8 %) and average germination rates (49.4 and 57.6 %), we estimated the percentages of viable hybrid seedlings following mixed pollination and heterospecific pollination to be 2.0 and 0.8 %, respectively.

**Table 6.** Hybrid seedlings between *Taraxacum japonicum* and *T. officinale* obtained from the hand pollination experiments at OU in 2014

Treatment	Seeds sown (no. of mothers)	No. of seedlings	No. of seedlings providing leaf samples	No. of seedlings identified as hybrids	Percentage of hybrid seedlings (%)
Natural pollination	621 (16)	264	255	0	0.0
Mixed pollination	740 (16)	304	224	16	7.1
Heterospecific pollination	190 (15)	96	96	10	10.4

## DISCUSSION

Our study results revealed that the reproductive success of the native *T. japonicum* at our study sites was unlikely to be associated with alien density or a pollinator preference for alien inflorescences, but was more likely to be associated with heterospecific pollen deposition from the alien species. The results also suggest that hybridization between the native and alien species might be a rare event.

Our field survey results did not show a negative effect of alien species density on native seed set. According to Feinsinger *et al.* (1991), if increases in flower density of one plant species lead to declining reproduction in another species with which it shares pollinators, then the plants might be competing for pollinators, or conspecific pollen carried by the shared pollinators might be lost. Their results also indicated that these interspecific effects on fertilization in self-incompatible plants (such as *T. japonicum*) might closely reflect nearby flower densities. Considering their findings, we infer that reproductive success of the native *T. japonicum* was not affected by competition with the alien flowers for pollinators or by conspecific pollen loss at our study sites. Our pollinator visitation observation results support this inference, although these observations must be considered preliminary because they were conducted over only a short period and in a different year from our field surveys.

Pollinator preference should not be excluded, however, as a possible effect of the alien on the native *Taraxacum* species. Kandori *et al.* (2009) observed pollinator behaviour in another region of Japan and found that at three of four locations, the pollinators preferred the alien *T. officinale* to the native *T. japonicum*. Moreover, reproductive success of the native species was lower when the alien species was present, and it was lowest when the density of the alien was highest. Although the presence of the aliens might have reduced the reproductive performance of the natives through not only pollinator preference but also heterospecific pollen deposition in their study, the density dependence of the reduction in native performance suggests pollinator preference rather than heterospecific pollen deposition, because the latter would have a frequency-dependent effect (Takakura *et al.*, 2009). The pollinator visitation observations of Kandori *et al.* (2009) together with the density dependence of the effect are consistent with the findings of Feinsinger *et al.* (1991). According to Kandori *et al.* (2009), greater nectar production by the alien species accounted for the pollinator preference for the alien species, but it did not explain why pollinators visited native flowers more frequently at one of their four locations. We did not measure nectar in our study, so further investigation is necessary to explain why our results differed from those of Kandori *et al.* (2009).

Considering our results together with those of Kandori *et al.* (2009), we can infer that pollinator preference may be a major mechanism of competition for pollination, but its importance may differ among regions, especially in plants such as *Taraxacum* species, which are visited by different pollinator species depending on the site. Heterospecific pollen deposition, on the other hand, might occur wherever species pairs, such as *Taraxacum* species pairs, share similar flower morphology, regardless of the region. Pollinator preference and heterospecific pollen deposition may have different ecological and evolutionary consequences for the species involved, although the two mechanisms are not exclusive, as shown by Brown *et al.* (2002). The consequences of competition for pollination observed in the field might therefore reflect a balance between them. In fact, at our study sites, the positive effects of a pollinator preference for the native species might counter the negative effects of alien pollen deposition, thus promoting the stability of the native population dynamics.

Our hand pollination experiments revealed that heterospecific pollen deposition reduced seed set of the native *T. japonicum*. Average seed set decreased by 1.4–13.3 % following simultaneous mixed pollination compared with that following conspecific pollination (Table 4). The results of our mixed pollination experiments that varied the order of application suggest that heterospecific pollen had a deleterious effect on seed set in the native species; average seed set was lowest when heterospecific pollen was applied first, whereas seed set did not decrease significantly when conspecific pollen was applied first (Table 5). The most plausible mechanism reducing reproductive success in *Taraxacum* may be usurpation or pre-emption of ovules by heterospecific pollen, as indicated by an examination of pollen tube growth (Nishida *et al.*, 2014).

Because we applied donor pollen in an amount that we supposed to be more than sufficient compared with the pollen amount received during natural pollination, the possibility that the results of our staggered mixed pollination experiments were actually an artefact cannot be ruled out. If the amount of pollen applied during the initial treatment was too large, the pollen applied 4 h later might have been unable to make sufficient contact with the stigmatic surface. However, Brock (2009) conducted staggered hand pollinations in which heterospecific pollination was followed 15 min later by conspecific pollination (apparently with pollen amounts similar to those used in our pollination experiments), and reported that seed set was not significantly different from that obtained by only conspecific pollination. Furthermore, in both the present study and an earlier study (Matsumoto *et al.*, 2010), seed set was reduced by simultaneous mixed pollination even though the conspecific pollen was applied just before the heterospecific pollen. Taken together, these findings support our inference that artificial stigma clogging cannot completely account for the reduced seed set in our mixed pollination experiments. Waser and Fugate (1986) and Caruso and Alfaro (2000), who conducted similar experiments in which the order of conspecific and heterospecific pollen application differed, reported that heterospecific pollen can severely affect the reproductive success of the focal species when it is applied earlier than conspecific pollen.

We consider allelopathic inhibition of conspecific pollen by the heterospecific pollen to be less likely to be a plausible mechanism reducing seed set in our species, because with that mechanism reproductive success following mixed pollination should have been reduced much more. For example, pollen germination of seven species in culture with pollen clusters of *Parthenium hysterophorus* was reduced by 62.9–100.0 % compared with the control through pollen allelopathy (Sukhada and Jayachandra, 1980); similarly, seed set of *Diervilla lonicera* was reduced by approx. 80 % following mixed pollination with approx. 20 % pollen of *Hieracium floribundum* (Thomson *et al.*, 1981). In certain plants, for example, *Erythronium grandiflorum* and *Erysimum capitatum*, pollen of *T. officinale* appears to have some allelopathic effects (Loughnan *et al.*, 2014). To clarify the possible allelopathic effects of *T. officinale* pollen, more trials with different plants are needed.

Hybridization is potentially an important mechanism of reproductive interference because it can not only reduce the fecundity of the parental taxa through ovule usurpation but can also influence recruitment and establishment of the parental taxa through competition for a suitable habitat (Burgess and Husband, 2006). In our results, hybridization between native and alien *Taraxacum* rarely resulted in viable seeds. Moreover, Morita *et al.* (1990) showed that most of the offspring resulting from hybridization experiments between diploid and triploid *Taraxacum* species were actually diploids produced by self-fertilization (mentor effect), and Mitsuyuki *et al.* (2014) reported only one confirmed hybrid among 430 tested plants grown from diploid *Taraxacum* seeds. Most *T. officinale* plants in western Japan are actually hybrids between *T. officinale* and the native diploid; Shibaike *et al.* (2002) have reported some genetic variation among the hybrids. Therefore, hybridization clearly occurs at least occasionally under natural conditions. Considering the results of the present study and those of Morita *et al.* (1990) and Mitusyuki *et al*. (2014), however, it is possible that most hybrids seen in the field might be a consequence of the clonal reproduction of rarely occurring natural hybrids. With regard to competition for suitable habitat between hybrids and parental taxa, Takakura *et al.* (2012) showed that the annual death rate of alien (presumably mostly hybrid) plants ranged from 0.27 to 0.50, about twice the annual rate of *T. japonicum* (0.10–0.29); this result suggests that the hybrids may not be as well suited to the habitat as the native species.

### Conclusion

Although our study pointed to heterospecific pollen deposition from the alien to the native *Taraxacum* species as the major mechanism of reproductive interference exerting a deleterious effect on the native species, other mechanisms (e.g. pollen allelopathy) should be further investigated to gain a comprehensive understanding of reproductive interference in *Taraxacum*. Additionally, conspecific pollen loss due not only to pollinator preference but also to pollen discounting should be evaluated from the standpoint of both female and male reproductive success (Muchhala and Thomson, 2012). In acknowledgment of these considerations, we suggest that the magnitude of the effect of each mechanism on the population dynamics should be evaluated to determine the mechanism’s impact on the temporal and spatial structure of plant communities and its influence on divergence of reproductive strategies. Simulations such as those conducted by Waser (1978), using parameter values derived from field and experimental studies, would help us to assess the importance of reproductive interference in ecology and evolution.

This study focused on the interaction between alien and native *Taraxacum* plants, but this system might be exceptional because the alien species, which is polyploid and apogamous, does not suffer reciprocally from reproductive interference. Heterospecific pollen deposition from a species that is both native and sexual might have a deleterious effect on another native species, however, insofar as reproductive interference occurs between them. Many existing studies of reproductive interference were conducted not in natural settings but in the laboratory (Gröning and Hochkirch, 2008; Kyogoku, 2015), or they examined interactions between alien and native species (e.g. Harder *et al.*, 1992; Burgess *et al.*, 2008; Takakura *et al.*, 2009). Some researchers may consider these experimental studies to overestimate heterospecific pollen deposition, which may be less common in nature. For example, on the basis of a literature review, Morales and Traveset (2008) reported that a reduction of conspecific pollen deposition is more commonly an outcome of pollinator sharing than of heterospecific pollen deposition, at least in animal-pollinated plants, and they inferred that the impact on plant reproduction of reduced conspecific pollen deposition mediated by conspecific pollen loss might be more deleterious than that of heterospecific pollen deposition. Why has a strong effect of heterospecific pollen deposition rarely been found between native species in nature? We suggest that it is because the effect is frequency dependent; as a result, one member of the species pair is likely to be rapidly excluded through a positive feedback effect on the population dynamics (Levin and Anderson, 1970; Takakura *et al.*, 2009). If the effect of heterospecific pollen deposition between a species pair is bi-directional and asymmetric, with the severity of the effect being greater in one direction than in the other, then the species causing the more severe effect reduces the number of offspring produced by the other species, thus becoming relatively more abundant in the next generation and having a more severe effect compared with that in the previous generation. Because of this positive feedback, Kyogoku (2015) identified heterospecific pollen deposition as a mechanism of demographic reproductive interference. In a theoretical study, Nishida *et al.* (2015) showed that two species that exert strong reproductive interference on each other cannot locally coexist on an evolutionary time scale even if the interacting species have equal per capita interference strength, although their eventual distributions also depend on differences in habitat suitability and in resource competition between the species. This inability to coexist locally may be one reason why we rarely observe a severe deleterious effect of heterospecific pollen deposition from one native plant species to another. As DeBach (1966) suggested, exclusion (displacement) of a species through competition that may have occurred in past years is largely unascertainable because the species has disappeared, but detailed studies will lead to the more frequent recognition of such exclusion, which is probably occurring more often because of the transport of organisms, either intentionally or accidentally, by human activities. Thus, to improve understanding of the ecological role of heterospecific pollen deposition, knowledge obtained from observations of alien–native interactions can be used to evaluate the impact of heterospecific pollen deposition in nature.

## FUNDING

This work was supported by JSPS KAKENHI grants (nos 22570088 and 26440211 to S.N., nos 21770041 and 24113510 to M.M.K., and no. 19770023 to K.-I.T.) from the Japan Society for the Promotion of Science and the Ministry of Education, Culture, Sports, Science and Technology, Japan.

## References

[CIT0001] Arceo-Gómez G, Ashman T-L 2011 Heterospecific pollen deposition: does diversity alter the consequences? New Phytologist 192: 738–746. 21777248 10.1111/j.1469-8137.2011.03831.x

[CIT0002] Bjerknes A-L, Totland Ø, Hegland SJ, Nielsen A 2007 Do alien plant invasions really affect pollination success in native plant species? *Biological Conservation* 138: 1–12.

[CIT0003] Brock MT 2009 Prezygotic barriers to gene flow between *Taraxacum ceratophorum* and the invasive *Taraxacum officinale* (Asteraceae). Oecologia 161: 241–251. 19504127 10.1007/s00442-009-1383-0

[CIT0004] Brown BJ, Mitchell RJ, Graham SA 2002 Competition for pollination between an invasive species (purple loosestrife) and a native congener. Ecology 83: 2328–2336.

[CIT0005] Burgess KS, Husband BC 2006 Habitat differentiation and the ecological costs of hybridization: the effects of introduced mulberry (*Morus alba*) on a native congener (*M. rubra*). Journal of Ecology 94: 1061–1069.

[CIT0006] Burgess KS, Morgan M, Husband BC 2008 Interspecific seed discounting and the fertility cost of hybridization in an endangered species. New Phytologist 177: 276–284. 17944826 10.1111/j.1469-8137.2007.02244.x

[CIT0007] Campbell DR 1986 Predicting plant reproductive success from models of competition for pollination. *Oikos* 47: 257–266.

[CIT0008] Caruso CM, Alfaro M 2000 Interspecific pollen transfer as a mechanism of competition: effect of *Castilleja linariaefolia* pollen on seed set of *Ipomopsis aggregata*. *Canadian Journal of Botany* 78: 600–606.

[CIT0009] Cock MJW 1978 The assessment of preference. Journal of Animal Ecology 47: 805–816.

[CIT0010] DeBach P 1966 The competitive displacement and coexistence principles. Annual Review of Entomology 11: 183–212.

[CIT0011] Eaton DAR, Fenster CB, Hereford J, Huang S, Ree RH 2012 Floral diversity and community structure in *Pedicularis* (Orobanchaceae). Ecology 93: S182–S194.

[CIT0012] Feinsinger P, Tiebout HM, Young BE 1991 Do tropical bird-pollinated plants exhibit density-dependent interactions? Field experiments. Ecology 72: 1953–1963.

[CIT0013] Gröning J, Hochkirch A 2008 Reproductive interference between animal species. The Quarterly Review of Biology 83: 257–282. 18792662 10.1086/590510

[CIT0014] Harder LD, Aizen MA 2010 Floral adaptation and diversification under pollen limitation. Philosophical Transactions of the Royal Society B: Biological Sciences 365: 529–543. 10.1098/rstb.2009.0226 PMC2838256 20047878

[CIT0015] Harder LD, Cruzan MB, Thomson JD 1992 Unilateral incompatibility and the effects of interspecific pollination for *Erythronium americanum* and *Erythronium albidum* (Liliaceae). *Canadian Journal of Botany* 71: 353–358.

[CIT0016] Hochkirch A, Gröning J, Bucker A 2007 Sympatry with the devil: reproductive interference could hamper species coexistence. *Journal of Animal Ecology* 76: 633–642. 17584368 10.1111/j.1365-2656.2007.01241.x

[CIT0017] Hoya A 2010 Evolution of hybridized dandelion. In: Muranaka T, Ishihama F, eds. Ecology of introduced organisms: adaptive evolution into new environments and possible counter measures. Tokyo: Bun-ichi Co. Ltd., 217–246 [in Japanese].

[CIT0018] Kandori I, Hirao T, Matsunaga S, Kurosaki T 2009 An invasive dandelion unilaterally reduces the reproduction of a native congener through competition for pollination. *Oecologia* 159: 559–569. 19153768 10.1007/s00442-008-1250-4

[CIT0019] Kyogoku D 2015 Reproductive interference: ecological and evolutionary consequences of interspecific promiscuity. Population Ecology 57: 253–260.

[CIT0020] Levin DA, Anderson WW 1970 Competition for pollinators between simultaneously flowering species. America Naturalist 104: 455–467.

[CIT0021] Loughnan D, Thomson JD, Ogilvie JE, Gilbert B 2014 *Taraxacum officinale* pollen depresses seed set of montane wildflowers through pollen allelopathy. *Journal of Pollination Ecology* 13: 146–150.

[CIT0022] Manly BFJ, Miller P, Cock MJW 1972 Analysis of a selective predation experiment. American Naturalist 106: 719–736.

[CIT0023] Matsumoto T, Takakura KI, Nishida T 2010 Alien pollen grains interfere with the reproductive success of native congener. Biological Invasions 12: 1617–1626.

[CIT0024] Mitchell RJ, Flanagan RJ, Brown BJ, Waser NM, Karron JD 2009 New frontiers in competition for pollination. Annals of Botany 103: 1403–1413. 19304814 10.1093/aob/mcp062 PMC2701753

[CIT0025] Mitsuyuki C, Hoya A, Shibaike H, Watanabe M, Yahara T 2014 Formation of a hybrid triploid agamosperm on a sexual diploid plant: evidence from progeny tests in *Taraxacum platycarpum* Dahlst. Plant Systematics and Evolution 300: 863–870.

[CIT0026] Morales CL, Traveset A 2008 Interspecific pollen transfer: magnitude, prevalence and consequences for plant fitness. Critical Reviews in Plant Sciences 27: 221–238.

[CIT0027] Morita T 1980 Dandelions. In: Hotta M, ed. Plant life histories. Tokyo: Heibonsha Limited, Publishers, 58–67.

[CIT0028] Morita T 1997 *Taraxacum officinale*: a compilospecies spread over the world. In: Yamaguchi H, ed. Natural history of weeds. Sapporo: Hokkaido University Publisher, 192–208.

[CIT0029] Morita T, Menken SBJ, Sterk AA 1990 Hybridization between European and Asian dandelions (*Taraxacum* section *Ruderalia* and section *Mongolica*). *New Phytologist* 114: 519–529. 10.1111/j.1469-8137.1990.tb00420.x 33873962

[CIT0030] Muchhala N, Thomson JD 2012 Interspecific competition in pollination systems: costs to male fitness via pollen misplacement. *Functional Ecology* 26: 476–482.

[CIT0031] Nishida S, Takakura KI, Nishida T, Matsumoto T, Kanaoka MM 2012 Differential effects of reproductive interference by an alien congener on native *Taraxacum* species. Biological Invasions 14: 439–447.

[CIT0032] Nishida S, Kanaoka MM, Hashimoto K, Takakura KI, Nishida T 2014 Pollen–pistil interactions in reproductive interference: mechanism driving the exclusive distribution of alien and native species of *Taraxacum*. Functional Ecology 28: 450–457.

[CIT0033] Nishida S, Hashimoto K, Kanaoka MM, Takakura KI, Nishida T 2017 Variation in the strength of reproductive interference from an alien congener to a native species in *Taraxacum*. Journal of Plant Research 130: 125–134. 27659681 10.1007/s10265-016-0865-5

[CIT0034] Nishida T, Takakura KI, Iwao K 2015 Host specialization by reproductive interference between closely related herbivorous insects. Population Ecology 57: 273–281.

[CIT0035] Ogawa K, Mototani I 1985 Invasion of the introduced dandelions and survival of the native ones in the Tokyo metropolitan area of Japan. Japanese Journal of Ecology 33: 443–452.

[CIT0036] R Core Team 2014 R: a language and environment for statistical computing. Vienna, Austria: R Foundation for Statistical Computing.

[CIT0037] Richard AJ 1970 Eutriploid facultative agamospermy in *Taraxacum*. *New Phytologist* 69: 761–774.

[CIT0038] Richardson BJ, Baverstock PR, Adams M 1986 Allozyme electrophoresis. San Diego: Academic Press.

[CIT0039] Robertson C 1895 The philosophy of flower seasons, and the phaenological relations of the entomophilous flora and the anthophilous insect fauna. American Naturalist 29: 97–117.

[CIT0040] Shibaike H, Akiyama H, Uchiyama A, Kasai K, Morita T 2002 Hybridization between European and Asian dandelions (*Taraxacum* section *Ruderalia* and section *Mongolica*) 2. Natural hybrids in Japan detected by chloroplast DNA marker. Journal of Plant Research 115: 321–328. 12579356 10.1007/s10265-002-0045-7

[CIT0041] Shiraishi S 1988 Inheritance of isozyme variation in Japanese black pine, *Pinus thumbergii* Parl. Silvae Genetica 37: 93–100.

[CIT0042] Sukhada DK, Jayachandra. 1980 Pollen allelopathy – a new phenomenon. *New Phytologist* 84: 739–746.

[CIT0043] Takakura KI, Nishida T, Matsumoto T, Nishida S 2009 Alien dandelion reduces the seed set of a native congener through frequency dependent and one-sided effects. Biological Invasions 11: 973–981.

[CIT0044] Takakura KI, Matsumoto T, Nishida T, Nishida S 2011 Effective range of reproductive interference exerted by an alien dandelion, *Taraxacum officinale*, on a native congener. Journal of Plant Research 124: 269–276. 20676914 10.1007/s10265-010-0368-8

[CIT0045] Takakura KI, Matsumoto T, Nishida S, Nishida T 2012 Analyses of the reproductive interference between native and invasive dandelions with individual based model. *Japanese Journal of Ecology* 62: 255–265 [in Japanese].

[CIT0046] Thomson JD, Andrews B, Plowright RC 1981 The effect of a foreign pollen on ovule development in *Diervilla lonicera* (Caprifoliaceae). New Phytologist 90: 777–783.

[CIT0047] Tsumura Y, Tomaru N, Suyama N, Na’eim M, Ohba K 1990 Laboratory manual of isozyme analysis. Bulletin of Tsukuba University Forests 6: 63–95 [in Japanese].

[CIT0048] Waser NM 1978 Interspecific pollen transfer and competition between co-occurring plant species. Oecologia 36: 223–236. 28309130 10.1007/BF00349811

[CIT0049] Waser NM 1983 Competition for pollination and floral character differences among sympatric plant species: a review of evidence. In: Jones CE, Little RJ, eds. Handbook of experimental pollination biology. New York: Van Nostrand Reinhold Company Inc., 277–293.

[CIT0050] Waser NM, Fugate ML 1986 Pollen precedence and stigma closure: a mechanism of competition for pollination between *Delphinium nelsonii* and *Ipomopsis aggregata*. *Oecologia* 70: 573–577. 28311501 10.1007/BF00379906

[CIT0051] Wolfinger R, O’Connell M 1993 Generalized linear mixed models: a pseudo-likelihood approach. *Journal of Statistical Computation and Simulation* 4: 233–243.

